# Development of Low-Frequency Phased Array for Imaging Defects in Concrete Structures

**DOI:** 10.3390/s21217012

**Published:** 2021-10-22

**Authors:** Yoshikazu Ohara, Kosuke Kikuchi, Toshihiro Tsuji, Tsuyoshi Mihara

**Affiliations:** Department of Materials Processing, Tohoku University, Aoba 6-6-02, Aoba-ku, Sendai 980-8579, Japan; kosuke.kikuchi.p1@gmail.com (K.K.); toshihiro.tsuji.b6@tohoku.ac.jp (T.T.); tsuyoshi.mihara.b8@tohoku.ac.jp (T.M.)

**Keywords:** low-frequency phased array, ultrasonic imaging, concrete, crack-type defect, delamination

## Abstract

The nondestructive inspection of concrete structures is indispensable for ensuring the safety and reliability of aging infrastructures. Ultrasonic waves having a frequency of tens of kHz are frequently used to reduce the scattering attenuation due to coarse aggregates. Such low frequencies enable the measurement of the thickness of concrete structures and detection of layer-type defects, such as delamination, whereas it causes a lack of sensitivity to crack-type defects. In this paper, to realize the ultrasonic phased array (PA) imaging of crack-type defects, we fabricated a low-frequency (LF) array transducer with a center frequency of hundreds of kHz. To avoid the crosstalk between piezoelectric elements and dampen the vibration of each element, we adopted soft lead zirconate titanate (soft PZT) with a low mechanical quality factor. Subsequently, we optimized the geometry of each piezoelectric element using a finite element method to generate a short pulse. After validating the design in a fundamental experiment using a single-element transducer, we fabricated a 32-element array transducer with a center frequency of 350 kHz. To show the imaging capability of the LF array transducer, we applied it to a concrete specimen with a delamination. As a result, the PA with the LF array transducer clearly visualized the delamination, which could not be visualized using the PA with a 2.5 MHz array transducer. Furthermore, we applied it to a more challenging defect, a slit, which is sometimes used to simulate crack-type defects. As a result, the PA with the LF array transducer clearly visualized a slit of 1 mm width and 40 mm height in a concrete specimen. Thus, we demonstrated the usefulness of the LF array transducer for inspecting crack-type defects.

## 1. Introduction

The aging of concrete infrastructures, such as bridges, highways, and tunnels, is a vital problem worldwide. Nondestructive testing (NDT) is a key technology for ensuring safety and reliability. One of the most widely used NDT methods for concrete structures is visual testing (VT). VT is a technique for inspecting surfaces. Another common NDT technique is the hammering test [1,2]. Layer-type defects, such as delaminations, in the vicinity of concrete surfaces can be detected from the sound reverberation following an impact of a hammer on concrete structures. These approaches are mainly employed to qualitatively inspect concrete subsurfaces.

Ultrasonic testing (UT) [3] is a promising approach for inspecting the interior of concrete structures. In UT, typically, a pulse-echo method using a transducer and/or a pitch-catch method with a pair of transducers are employed. Various ultrasonic wave propagation characteristics, such as velocity, amplitude, attenuation, and frequency, can be used to measure the thickness and detect the damage of concrete structures [3,4]. Nevertheless, such UT suffers insufficient accuracy in the quantitative measurement of internal defects in concrete structures.

Ultrasonic phased arrays (PAs) [5,6] are powerful tools for visualizing solids. PAs were originally developed for medical diagnosis [7] and have also been widely adopted to visualize the internal defects of metals for industrial applications. The recent progress in PAs for the inspection of metals is noteworthy [8,9,10,11,12,13,14]. PAs typically use a linear array transducer composed of multiple rectangular piezoelectric elements that can act as both transmitters and receivers. PAs detect and locate defects from B-scan images obtained by exciting the array elements under a delay law so that the wavefront injected into a sample has the desired characteristics, such as beam steering and focusing, in arbitrary regions. Note that the sensitivity and resolution of PA imaging can theoretically become higher when using a higher frequency. However, one needs to consider the effect of attenuation. Hence, frequency on the order of MHz has mainly been used to inspect metals. On the other hand, such a frequency range cannot be used to inspect concrete structures since concrete is much more attenuative than metals. Hence, an array transducer with a sub-MHz frequency is indispensable for the PA imaging of concrete structures.

As one of the candidates for ultrasonic imaging of concrete structures, a low-frequency (LF) PA system, MIRA, using a dry-point-contact (DPC) array transducer is commercially available [15,16,17]. The DPC array transducer generates and receives shear-horizontal (SH) waves at a very low frequency of 55 kHz, which can successfully reduce the effect of the high attenuation of concrete structures. Its use for thickness measurement and delamination detections has been demonstrated [15,16,17]. However, the sensitivity of MIRA to vertical crack-type defects may be insufficient owing to the very low frequency (i.e., long wavelength). Given that the sensitivity can be improved by increasing the frequency, a PA using an LF array transducer with a center frequency of hundreds of kHz can be suitable for the inspection of crack-type defects in concrete structures.

**Figure 1:**
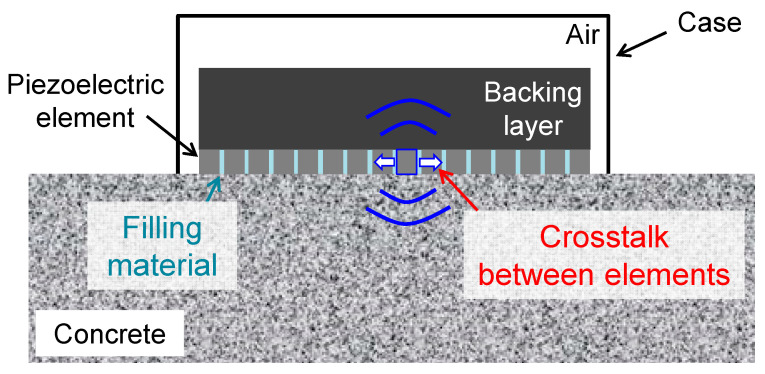
Structure of conventional array transducer with filling material and backing layer.

The fabrication of an LF array transducer with a center frequency of hundreds of kHz can encounter two difficulties if the LF array transducer has the conventional structure illustrated in Figure 1. The first difficulty is the crosstalk between piezoelectric elements [18,19]. This crosstalk is caused by the insufficient mechanical and electrical isolation between elements. For LF array transducers, the transmission of vibration modes to adjacent elements can be a dominant factor of the crosstalk owing to insufficient mechanical isolation. As shown in Figure 1, adjacent piezoelectric elements are physically connected via filling materials such as plastic resin. The excitation of piezoelectric elements of an array transducer generates not only thickness vibration but also lateral vibration. The former generates an ultrasonic wave in a sample and is used for PA imaging. The latter causes an ultrasonic wave that laterally propagates through filling materials to the adjacent elements in the array transducer. Note that LF ultrasonic waves are less attenuative than those with MHz frequencies. Therefore, the crosstalk due to the insufficient mechanical isolation between the elements can cause severe noise and reduce the capability to control the beam direction in transmission and reception [18]. The second difficulty is how to generate a short pulse, which is required for a high temporal resolution. A backing layer bonded on piezoelectric elements plays a vital role in dampening the MHz frequency vibration of the piezoelectric elements after their excitation [3]. This results in improved axial resolution and a broadened frequency bandwidth [20]. The backing layer also dissipates the backward energy to prevent the unwanted backward energy from returning to the piezoelectric materials. However, dampening the LF vibrations of piezoelectric elements is intrinsically difficult. Additionally, the attenuative materials, e.g., materials composed of rubber and heavy metallic particles, adequate for MHz frequencies [21] may be ineffective in suppressing the LF vibration that can reverberate in a backing layer. Such multiple reflections [22] in the backing layer can cause additional, long-standing noise. Thus, the structure (Figure 1) of a conventional array transducer with center frequencies on the order of MHz is unsuitable for an LF array transducer.

In this study, we propose a novel structure that can avoid the crosstalk between array elements and dampen the LF vibration of each element to achieve an LF array transducer with a center frequency of hundreds of kHz. We also optimize the geometry of piezoelectric elements using a finite element (FE) analysis to generate a short pulse. After validating the design in a fundamental experiment using a single-element transducer, we fabricated a 32-element LF array transducer. We demonstrate the imaging capability in concrete specimens with a delamination and slit. We also discuss possible future applications of the LF array transducer.

## 2. Development of LF Array Transducer

The use of a high frequency can enhance the resolution of PA images and the sensitivity to defects, whereas the effect of the attenuation increases with increasing the frequency. Given a balance between the attenuation and the resolution and sensitivity, the inspection of metals is carried out using ultrasound with a MHz frequency. On the other hand, the attenuation of concrete structures is much higher than that of metals. To inspect such highly attenuative materials, the use of a lower frequency is indispensable.

To realize an LF array transducer having a center frequency of hundreds of kHz, it is essential to avoid the crosstalk between piezoelectric elements and dampen the vibration of each element, as they can lead to the generation of a short pulse and a decrease of the dead zone that appears from the top surface to a certain depth in PA images. As illustrated in Figure 1, the piezoelectric elements are physically connected via filling materials, which can increase the breakdown voltage and mechanical stability. However, the filling material creates physical connections between adjacent piezoelectric elements in the lateral direction, causing severe crosstalk. The backing layer dampens the vibration of each piezoelectric element after their excitation, which can result in a short pulse [3,21,22]. However, the backing layer may not contribute to the generation of a short LF pulse since LF vibrations are less attenuative than high-frequency ones. This may also cause additional noise owing to the long-standing reverberation in the backing layer [22].

**Figure 2:**
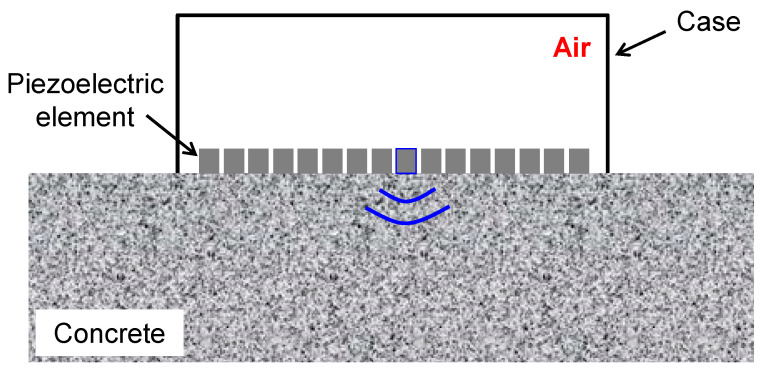
Proposed structure without filling materials or backing layer for LF array transducer.

To overcome the difficulties, we propose a structure without filling materials or a backing layer for LF array transducers. Figure 2 illustrates the structure proposed. By removing the filling materials and backing layer, the ultrasonic waves generated by firing piezoelectric elements propagate only in a contacted specimen since they cannot propagate in the array transducer because of a significant acoustic impedance mismatch between the piezoelectric elements and air. Note that the electrical isolation and mechanical stability of piezoelectric elements can also be guaranteed because the size of LF piezoelectric elements is larger than that of MHz array transducers. Thus, the problems of crosstalk and reverberation are simultaneously avoidable.

On the other hand, the lack of a backing layer can cause a low temporal resolution for transmitted and received signals, resulting in a deteriorated axial resolution and a narrower frequency bandwidth. To solve this problem, we adopted soft lead zirconate titanate (soft PZT) (C9, Fuji Ceramics, Fujinomiya, Japan) with a low mechanical quality factor *Q*_m_. Table 1 shows the characteristics of the soft PZT C9 and lead titanate (PT) (M6, Fuji Ceramics, Japan). Note that the PT was produced to selectively utilize thickness resonance. A low *Q*_m_ material has the characteristic of dampening the vibration by itself. In addition, such a material has a high longitudinal piezoelectric coefficient *d*_33_, resulting in a large-displacement incident wave. Nevertheless, such a soft PZT was not expected to be effective as a piezoelectric material of ultrasonic transducers for two reasons.

The first reason is the difficulty of exciting the soft PZT with typical 50 W pulsers. Soft PZT has high relative permittivity e_33_/e_0_, where e_33_ and e_0_ are the soft PZT and vacuum permittivities, respectively. The electrical impedance of the transducer made of the soft PZT tends to be low. Hence, a special high-current pulser would be required to effectively excite a MHz-frequency monolithic transducer [23,24]. On the other hand, the low frequency and small size of array elements would have a higher electrical impedance. This enables us to excite the soft PZT piezoelectric elements with the pulsers of standard PA systems.

The second reason is the strong lateral vibration due to the high transverse piezoelectric coefficient *d*_31_. When using the thickness resonance of piezoelectric materials, *d*_33_ is the most important property. The *d*_33_ of soft PZT is approximately 10 times as high as that of PT, whereas the *d*_31_ of soft PZT is approximately 100 times as high as that of PT. The fundamental resonance frequency of the lateral mode is typically lower than that of the longitudinal (thickness) mode. However, some of the higher-order resonance frequencies can exist around the fundamental resonance frequency of the thickness mode. Note that standard PA systems have pulsers that can generate a short pulse or one-cycle square wave as an excitation voltage. Such input voltages have a broad bandwidth and, therefore, can simultaneously excite multiple resonance modes. The superposition of the multiple lateral modes and fundamental thickness mode can significantly lower the temporal resolution of transmitted and received signals.

To generate a short pulse using the soft PZT, we propose utilizing a coupled resonance between the thickness and lateral vibrations. The lateral and thickness resonance frequencies are determined by the geometry of the soft PZT element. When the fundamental resonance frequency of the thickness mode is sufficiently far from that of the fundamental lateral mode, they exist as independent resonance modes. When the resonance frequencies become close to each other for a certain geometry of the soft PZT element, they are not independent and are complexly coupled. It can be expected that utilizing appropriate coupled resonance modes leads to a high temporal resolution for transmitted and received signals.

To optimize the geometry of the soft PZT elements, we performed a 2D FE simulation using the software PZFlex [25,26] based on the coupled piezoelectric–vibration analysis. The parameters of the soft PZT C9 used in the FE simulation are presented in Table 2. We varied the element width as 8, 6, 4, 3, and 2 mm for a fixed thickness of 4 mm, where the Courant number was 0.9. When the element width was much larger than the thickness of 4 mm, the thickness resonance frequency was 500 kHz.

**Figure 3:**
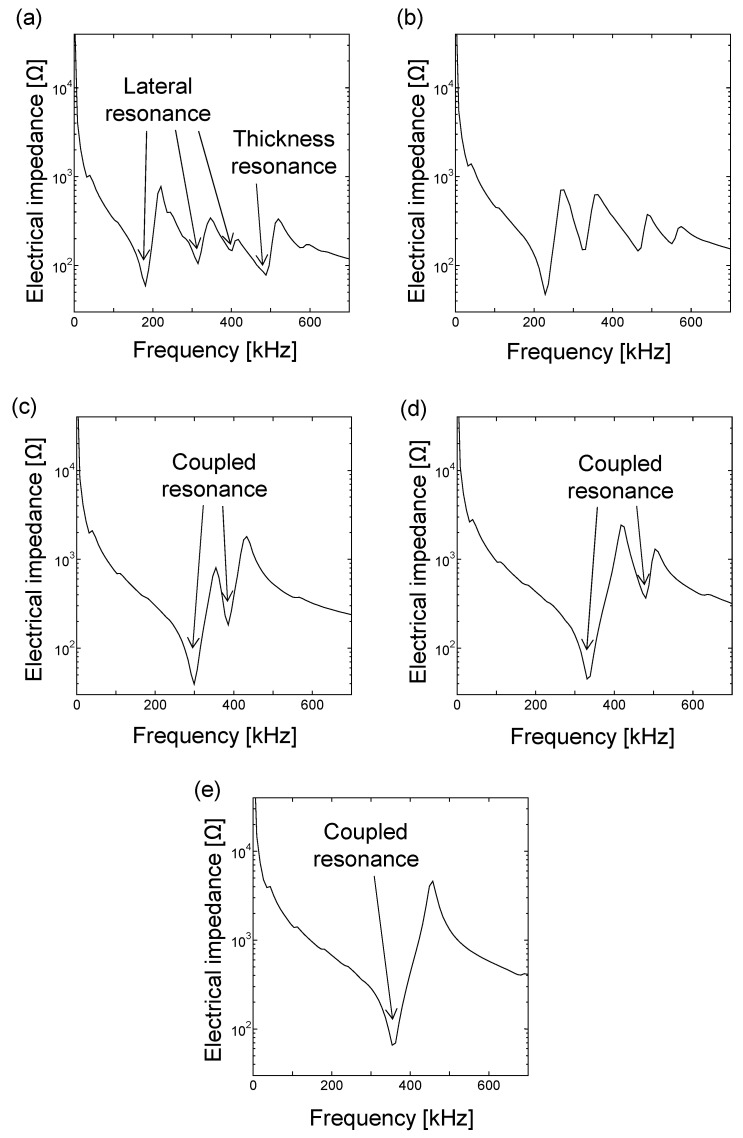
Electrical impedance calculated by 2D FE analysis with various element widths and a fixed thickness of 4 mm. Element width: (**a**) 8 mm, (**b**) 6 mm, (**c**) 4 mm, (**d**) 3 mm, and (**e**) 2 mm.

Figure 3 shows the electrical impedance calculated by the 2D FE simulation. At the element width of 8 mm (Figure 3a), multiple resonance peaks were observed. The thickness resonance appeared to be approximately 500 kHz, as expected. The other lower resonance peaks correspond to the fundamental and higher-order modes of the lateral resonances. This shows that each resonance mode existed independently at the element width of 8 mm. On the other hand, as the element width decreased from 6 mm to 3 mm, the resonance modes were complexly coupled, as shown in Figure 3b–d. At the element width of 2 mm, a single resonance mode appeared at approximately 350 kHz. The utilization of the single resonance mode can improve the axial resolution and broaden the frequency bandwidth. Although it can be expected that a smaller element width also shows a single resonance peak, the footprint of each piezoelectric element should be large to maximize the energy of the incident waves. Therefore, we selected the cross-section of the piezoelectric elements having the aspect ratio of 2 (i.e., 4 mm thickness and 2 mm width).

**Figure 4:**
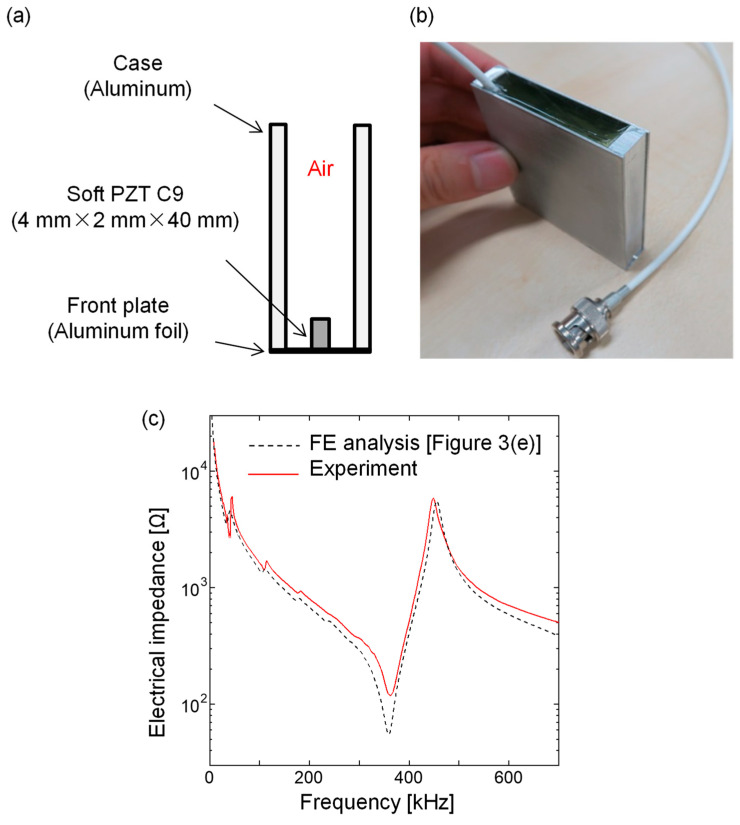
Transducer composed of a single piezoelectric (C9) element with 4 mm thickness and 2 mm width and the spectra of the electrical impedance. (**a**) Schematic illustration; (**b**) photograph of the fabricated transducer; (**c**) electrical impedance spectra.

To validate the FE analysis, we fabricated a transducer composed of a single element (soft PZT C9), adopting the design obtained by 2D FE simulation, as shown in Figure 4a,b. Here, the length of the piezoelectric element was 40 mm to make it a rectangular shape for a linear array transducer. We measured the electrical impedance of the fabricated transducer with an impedance meter. As a result, the resonance spectrum was found to be in good agreement with the prediction by the FE analysis, as shown in Figure 4c.

**Figure 5:**
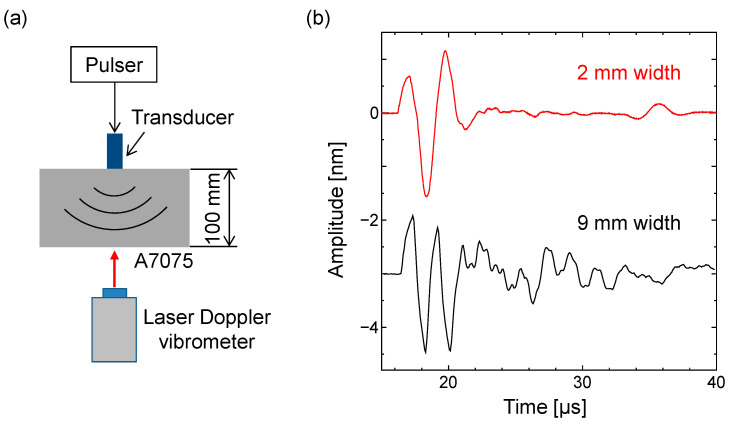
Transmitted waves of the 2 mm and 9 mm wide transducers. (**a**) Schematic of the experimental setup; (**b**) transmitted waves measured at the bottom of an aluminum-alloy sample using a laser Doppler vibrometer.

Furthermore, we experimentally examined the usefulness of the fabricated transducer with a single resonance frequency. As illustrated in Figure 5a, the transmitted wave was measured at the bottom of an aluminum-alloy specimen with 100 mm thickness using a laser Doppler vibrometer (Polytec, OFV-505). For comparison, we also fabricated the transducer composed of a single element with 4 mm thickness and 9 mm width. The excitation voltage was a square wave with 100 V. For the 9 mm wide transducer, the multiple frequency components were observed in the lower waveform of Figure 5b, as expected from the results of the 2D FE simulation. The transmitted wave is unsuitable for PA imaging because of a considerably low temporal resolution of the transmitted wave. In contrast, for the 2 mm wide transducer, a single pulse was observed in the upper waveform of Figure 5b, which is suitable for PA imaging because of its excellent temporal resolution. This shows that the soft PZT transducer without a backing layer is useful for dampening the LF vibration of the piezoelectric element. Thus, we validated the geometry (4 mm × 2 mm × 40 mm) of the soft PZT elements for an LF array transducer.

**Figure 6:**
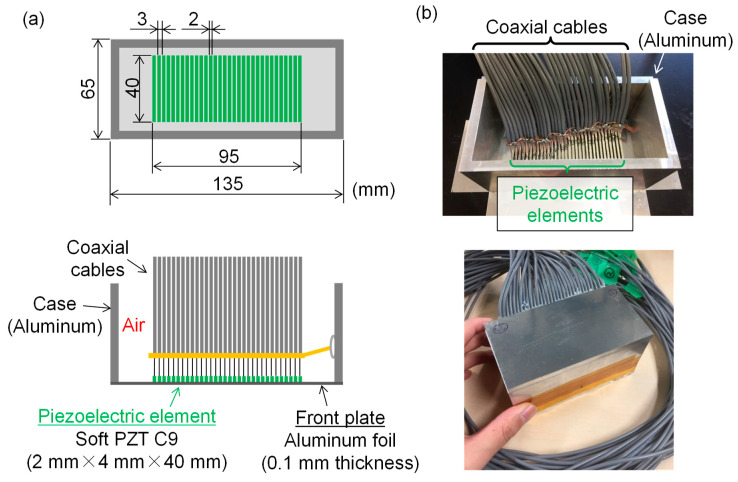
Fabrication of 32-element LF array transducer with the center frequency of 350 kHz. (**a**) Schematic showing the structure of LF array transducer; (**b**) photographs of the fabricated LF array transducer.

On the basis of the design of the soft PZT C9 element with 4 mm × 2 mm × 40 mm, we fabricated a 32-element LF array transducer. Figure 6a shows the structure of the LF array transducer. We adopted the structure without a backing layer or filling materials. We sliced the soft PZT (C9) plate (4 mm × 40 mm × 40 mm) into a rectangular shape (4 mm × 2 mm × 40 mm) using a dicing machine. The element pitch was selected to be 3 mm to avoid generating grating lobes. After bonding the 32 piezoelectric rectangles on the front plate made of 0.1 mm thick aluminum foil, we wired each element to a coaxial cable. The aluminum case was also bonded on the front plate. Figure 6b shows the fabricated LF array transducer.

**Figure 7:**
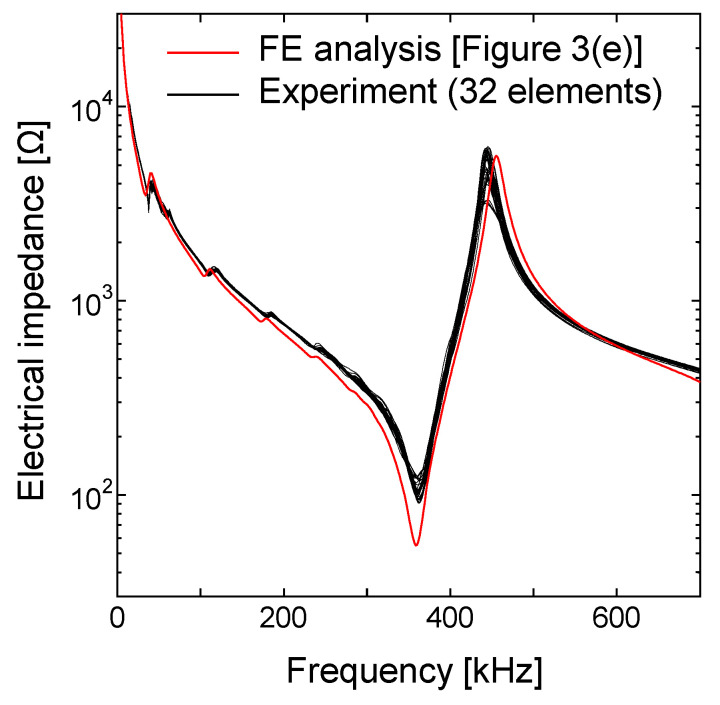
Electrical impedance spectra obtained by experiment and 2D FE simulation. The black curves represent the electrical impedance spectra of all of the fabricated LF array elements. The red curve represents the electrical impedance spectrum (Figure 3e) predicted by 2D FE simulation.

To confirm the uniformity of the 32 elements of the fabricated LF array transducer, we measured the electrical impedance of all elements. Figure 7 shows the electrical impedance spectra measured with an impedance meter. All electrical impedances were in excellent agreement with the FE simulation results (Figure 3e). This shows the high precision of the fabrication process.

## 3. Experiments of Imaging Concrete Specimens

### 3.1. Concrete Specimen with a Delamination

**Figure 8:**
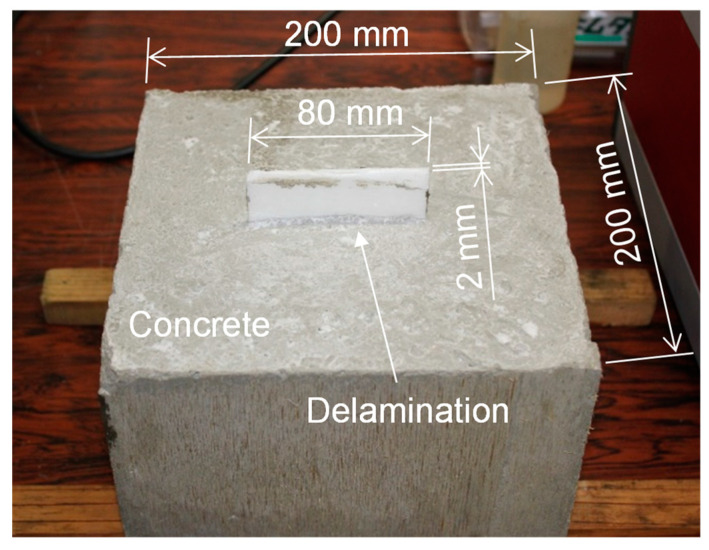
Concrete specimen with a delamination simulated with a Styrofoam plate (2 mm thickness, 80 mm width).

To demonstrate the effectiveness of the LF array transducer, we made a concrete specimen with a size of 200 mm × 200 mm × 135 mm and a delamination (Figure 8). Delamination in concrete structures is generated because of corrosive environments and repeated loadings. However, it may not be easy for us to make a delamination by simulating such conditions in a laboratory. On the other hand, the delamination can be regarded as a defect with a thin air layer, which causes ultrasonic reflection and scattering because of the acoustic impedance mismatching between concrete and air [3]. Note that the acoustic impedance of a Styrofoam plate is much lower than that of concrete and can be approximated to air. Hence, a delamination can be simulated by embedding a Styrofoam plate in concrete structures [27,28]. Here, we made an artificial delamination with a Styrofoam plate of 2 mm thickness and 80 mm width inserted at a depth of 100 mm. The longitudinal wave speed of the concrete specimen was measured to be 3200 m/s.

**Figure 9:**
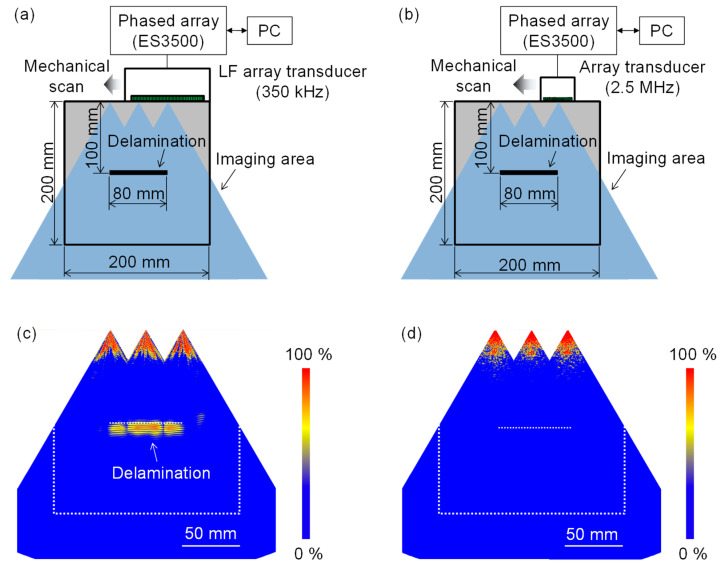
Experimental configurations for visualizing delamination in concrete specimen and imaging results obtained with LF and 2.5 MHz array transducers. (**a**,**b**) Experimental configurations for the LF and 2.5 MHz array transducers, respectively; (**c**,**d**) imaging results obtained with the LF and 2.5 MHz array transducers, respectively.

Figure 9a shows the experimental setup for imaging the concrete specimen with a delamination. We operated the LF array transducer using a PA controller (Hitachi, ES3500). The focal points were set between −30° and 30° with 1° steps at a fixed depth of 100 mm. As illustrated in Figure 9a, we mechanically scanned the LF array transducer on the top surface to visualize the whole delamination. For comparison, we also used a 32-element array transducer with a center frequency of 2.5 MHz and an element pitch of 1 mm (Figure 9b), which is commercially available as an array transducer with a relatively low center frequency for the inspection of metal.

Figure 9c,d are the PA imaging results obtained with the LF and 2.5 MHz array transducers, respectively, where we merged three images obtained at three measurement positions. When using the LF array transducer, the delamination was visualized with a high signal-to-noise ratio (SNR) in Figure 9c, from which the size and depth of the delamination can be accurately measured. The merged PA image has two local weak regions of the delamination response at the connecting area of the three PA images. Given that the PA imaging was performed in real time, a smoother delamination response can be obtained by selecting a smaller scan pitch. In contrast, the PA image (Figure 9d) obtained with the 2.5 MHz array transducer did not visualize the delamination. This can be explained by the much higher scattering attenuation of concrete structures than that of metals. Given that the scattering attenuation is strongly dependent on the ultrasonic frequency, it is reasonable that the delamination was invisible when using a frequency of 2.5 MHz. This implies that the commercial array transducer having a frequency in the MHz range is inapplicable to the inspection of concrete structures. Thus, the LF array transducer fabricated in this study successfully reduced the effect of the scattering attenuation due to aggregates.

### 3.2. Concrete Specimen with Crack-Type Defect (i.e., a Slit)

**Figure 10:**
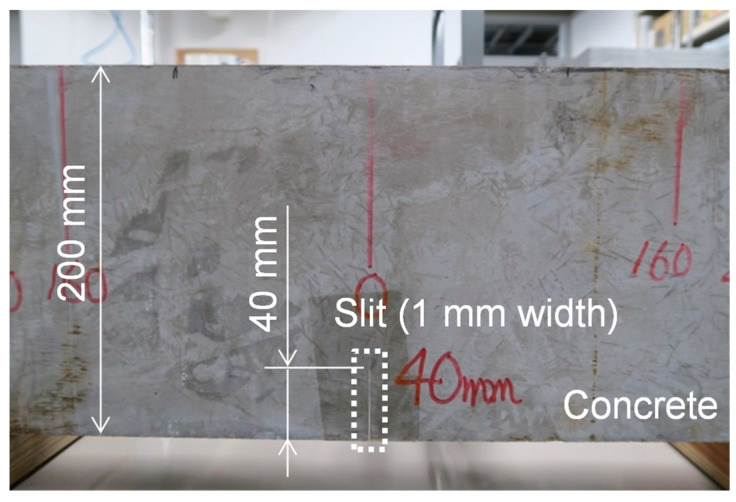
Concrete specimen with a slit (1 mm width, 40 mm height).

As a more challenging target, we prepared a concrete specimen with a crack-type defect (i.e., a slit), as shown in Figure 10. The specimen size was 200 mm × 200 mm × 800 mm. We machined a slit of 1 mm width and 40 mm height from the bottom. The longitudinal wave speed was measured to be 5000 m/s.

**Figure 11:**
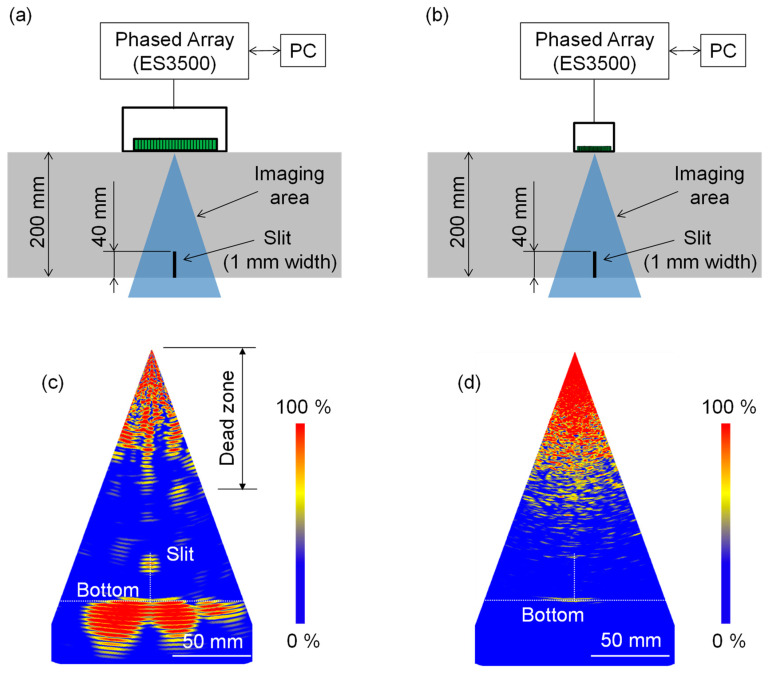
Experimental configurations for visualizing slit in concrete specimen and imaging results obtained with LF and 2.5 MHz array transducers. (**a**,**b**) Experimental configurations for the LF and 2.5 MHz array transducers, respectively; (**c**,**d**) imaging results obtained with the LF and 2.5 MHz array transducers, respectively.

Figure 11a,b show the experimental configurations for the LF and 2.5 MHz array transducers, respectively. We used the same PA equipment as that described in Section 3.1. The focal points were set to be from −20° to 20° with 0.5° steps in terms of angle and from 120 mm to 200 mm with 40 mm steps in terms of depth.

Figure 11c,d are the PA imaging results obtained with the LF and 2.5 MHz array transducers, respectively. When using the LF array transducer, a large dead zone appeared near the top surface. The dead zone is due to the ringing time of the electrically excited transducer and is regarded as an uninspectable region [3]. Despite of the same LF array transducer being used to obtain Figure 9c and Figure 11c, the dead zone in Figure 11c was much larger than that in Figure 9c. This is because of the difference in the receiver gain. Since the response of the slit was much weaker than that of the delamination, we increased the receiver gain by 10 dB. At the same time, this also amplified the dead zone, as shown in Figure 11c. Nevertheless, the slit was visualized below the dead zone with a high SNR, as shown in Figure 11c. The slit depth obtained from Figure 11c was in agreement with the actual depth of 40 mm. This shows that the frequency of 350 kHz was appropriate for reducing the effect of scattering attenuation and achieving the high sensitivity to the slit in this specimen. In contrast, the PA image (Figure 11d) obtained with the 2.5 MHz array transducer did not visualize the slit. In addition, even though it is much easier to visualize the bottom than the slit, the bottom response was very weak. This shows that most of the 2.5 MHz wave was attenuated before returning from the bottom. Thus, the difference in the bottom response between Figure 11c,d clearly shows the usefulness of utilizing the LF wave for highly attenuative materials. Furthermore, given the high SNR in Figure 11c, the PA using the LF transducer can be expected to visualize a narrower slit (or natural cracks), which will be an interesting future topic.

## 4. Discussion

We demonstrated the effectiveness of the LF array transducer in concrete specimens with a delamination and slit. In contrast to a conventional array transducer with a backing layer and filling material [29,30,31,32], the structure without a backing layer and filling materials and the shape optimization of the soft PZT element resulted in a suppressed dead zone in addition to reduced scattering attenuation. However, it is impossible to suppress the dead zone perfectly. For the inspection of the subsurface region in concrete structures, a surface-acoustic-wave phased array (SAW PA) [33,34,35] seems promising. The SAW PA uses a wedge to generate a Rayleigh wave via mode conversion. The penetration depth of Rayleigh waves is approximately one wavelength. The penetration depth can be changed by using a different frequency and mode. Although the SAW PA has been verified to be effective for the inspection of metals, it has yet to be applied to concrete inspections. The SAW PA using the LF array transducer would be an exciting topic for concrete inspection.

In this study, we carried out imaging experiments using incident longitudinal and scattered longitudinal waves. On the other hand, the mode conversion from the longitudinal wave to the transverse one occurs at scattering; this has been utilized for the PA imaging of metals [36,37,38,39,40,41]. The utilization of mode-converted scattered waves in the PA using the LF array transducer will provide further information on defects in concrete structures.

For the imaging of the concrete specimens, we used a typical PA imaging algorithm called delay and sum (DAS) processing [6,7]. The SNRs were sufficient in Figure 9c and Figure 11c. On the other hand, the imaging results may be affected by uncertainties because of the difference in the coupling gel between the LF array transducer and concrete specimens. To overcome such a problem, a fuzzy processing technique would be promising [42].

To achieve high-sensitivity imaging for concrete specimens, we selected a frequency of 350 kHz, which is relatively high for typical concrete UT. Therefore, such a frequency would be too high to inspect more attenuative concrete structures, e.g., those containing larger aggregates. The frequency selection depends on the balance between the attenuation of concrete structures and the sensitivity required to detect defects. It is essential to use a suitable frequency for each concrete structure. Note that the method for designing the structure of the LF array transducer proposed in this study is very general and can be directly applied to different frequencies. For example, to inspect more attenuative materials, one can make an LF array transducer with a lower resonance frequency by increasing the size of each piezoelectric element with the same shape since an ultrasonic wave with such a lower frequency can propagate a long distance while reducing the effect of attenuation. On the other hand, the lateral resolution ΔX of PA images is determined as follows [43]:

(1) $$\Delta X\propto \lambda \frac{d}{A},$$

where λ is the wavelength, d is the distance from the top surface, and A is the size of the aperture (i.e., the element pitch × the number of elements). Equation (1) shows that the use of a low frequency (i.e., a large λ) causes a low ΔX. In contrast, ΔX can be improved by selecting a large A, which can be realized by increasing the element pitch. However, an array transducer with an element pitch of greater than λ/2 causes the generation of grating lobes, resulting in severe artefacts [44]. To avoid grating lobes and obtain sufficient image resolution, the design of an LF array transducer should be optimized following the above discussion. On the other hand, the scheme proposed in this study can be used to design an LF 2D matrix array transducer [45,46] for 3D concrete imaging, which is an exciting topic of future work.

The classification of the type of defects is an important and challenging task. In this study, we measured two types of defects (i.e., a delamination and slit). The imaging results showed the difference in the lateral size in Figure 9c and Figure 11c, classifying the type of defects between the delamination and the slit. On the other hand, the image resolution is determined using Equation (1). Therefore, it may be difficult to classify a similar size of defects, such as slits and cracks, as well as defects smaller than the image resolution, such as porosities. To overcome the limitation of image resolution, super-resolution algorithms have been widely studied [47,48,49,50,51,52]. The combination of an LF phased array with such imaging algorithms is an exciting topic for classifying the type of defects.

## 5. Conclusions

In this paper, we proposed a novel structure for achieving an LF array transducer with a center frequency of hundreds of kHz for concrete inspection. To avoid the crosstalk between piezoelectric elements and to dampen the vibration of each element, we adopted a soft PZT (C9, Fuji Ceramics, Fujinomiya, Japan) with a low *Q*_m_ value and a structure without a backing layer or filling materials. Subsequently, we optimized the geometry of each piezoelectric element by FE analysis to generate a short pulse. After validating the design in a fundamental experiment using a single-element transducer, we fabricated a 32-element array transducer with a center frequency of 350 kHz. To show the imaging capability of the LF array transducer, we applied it to a concrete specimen with a delamination. As a result, the PA with the LF array transducer clearly visualized the delamination that could not be visualized using the PA with a 2.5 MHz array transducer. Furthermore, we applied it to a more challenging defect, a slit, which is sometimes used to simulate a crack-type defect. As a result, the PA using the LF array transducer clearly visualized a slit of 1 mm width and 40 mm height in the concrete specimen. Thus, we demonstrated the usefulness of the LF array transducer for inspecting crack-type defects in concrete structures.

## Figures and Tables

**Table 1:** Properties of piezoelectric materials.

Piezoelectric Material	ε_33_/ε_0_	*d*_33_ (×10^−12^ m/V)	*d*_31_ (×10^−12^ m/V)	*Q*_m_
PT (M6)	215	71	−3.7	850
Soft PZT (C9)	6640	718	−354	25

**Table 2:** Parameters of soft PZT C9 used for 2D FE simulation.

Parameter	Value	Parameter	Value
Geometry Thickness (mm) Width (mm)	4 8, 6, 4, 3, 2	Density (kg/m^3^)	7750
		Piezoelectric constants *d*_33_ (×10^−12^ m/V) *d*_31_ (×10^−12^ m/V) *d*_15_ (×10^−12^ m/V)	718 −354 827
Elastic constants *C*_11_ (GPa) *C*_33_ (GPa) *C*_55_ (GPa)	65 64 26		

## Data Availability

Not applicable.
